# Supplementary material for: International overview of sea turtle fibropapillomatosis: a survey of expert opinions and trends
Source: Front Cell Dev Biol. 2024 Aug 22;12:1445438. doi: 10.3389/fcell.2024.1445438 (PMC11374714; doi:10.3389/fcell.2024.1445438)

a

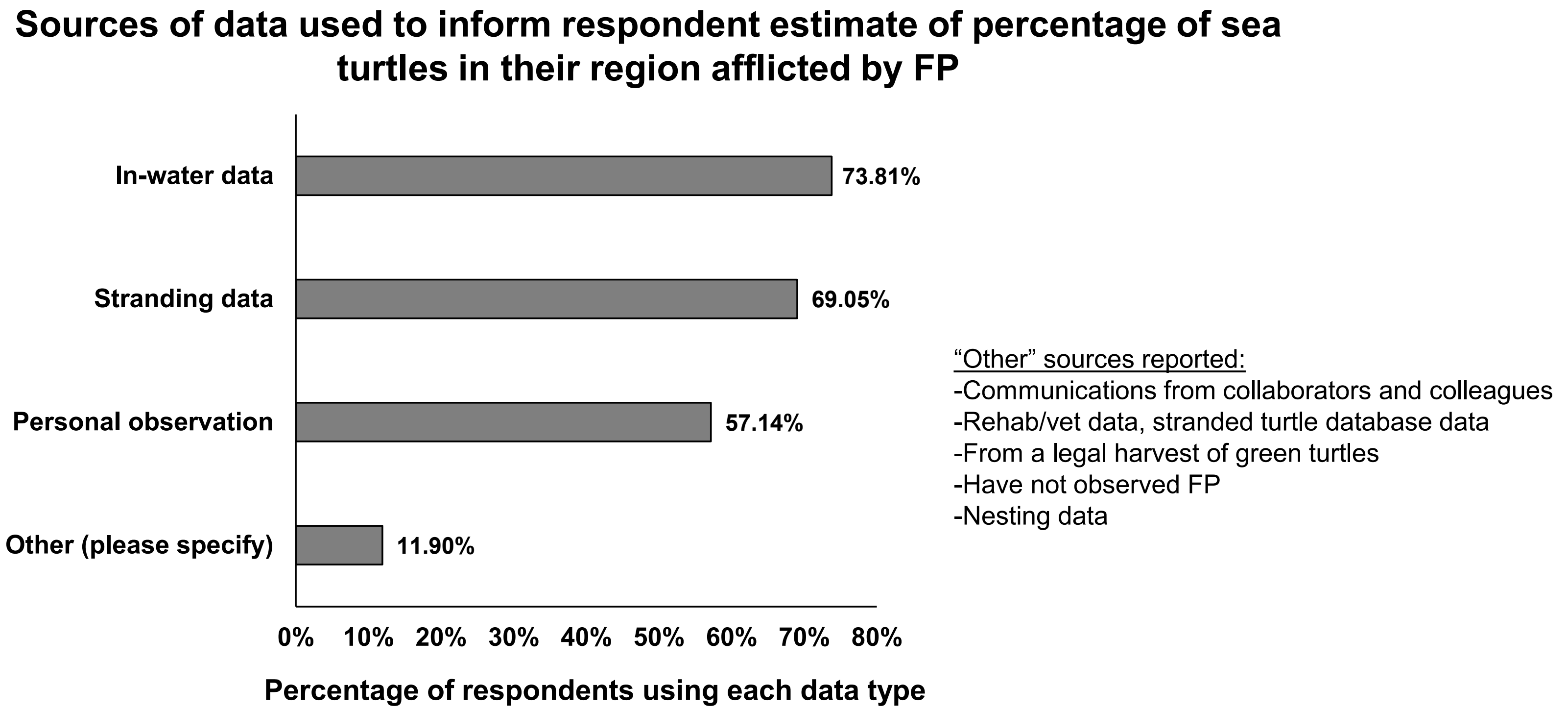
Sources of data used to inform respondent estimate of percentage of sea turtles in their region afflicted by FP
In-water data
73.81%
Stranding data
69.05%
Personal observation
57.14%
Other (please specify)
11.90%
0%
10%
20%
30%
40%
50%
60%
70%
80%
Percentage of respondents using each data type
"Other" sources reported:
-Communications from collaborators and colleagues
-Rehab/vet data, stranded turtle database data
-From a legal harvest of green turtles
-Have not observed FP
-Nesting data

b

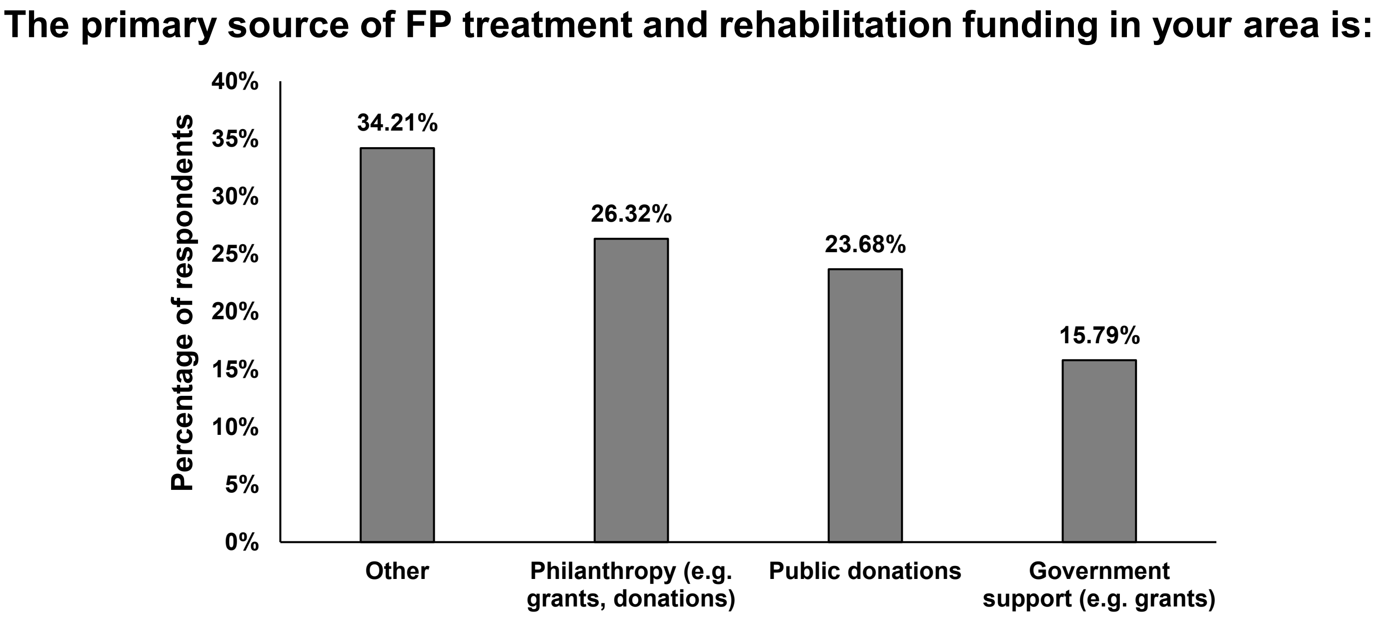
The primary source of FP treatment and rehabilitation funding in your area is:
Percentage of respondents
40%
35%
30%
25%
20%
15%
10%
5%
0%
34.21%
26.32%
23.68%
15.79%
Other
Philanthropy (e.g. grants, donations)
Public donations
Government support (e.g. grants)

c

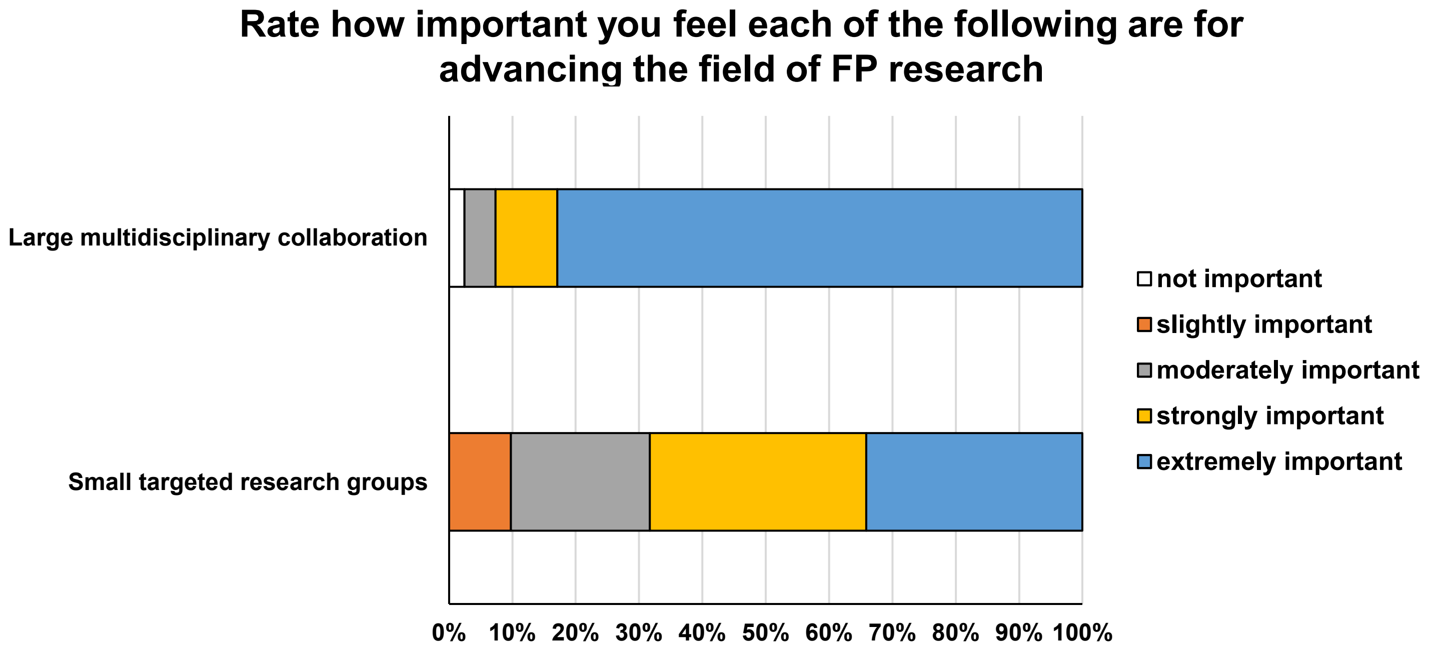
Rate how important you feel each of the following are for advancing the field of FP research
Large multidisciplinary collaboration
Small targeted research groups
0%
10%
20%
30%
40%
50%
60%
70%
80%
90%
100%
not important
slightly important
moderately important
strongly important
extremely important

Supplemental Figure 2

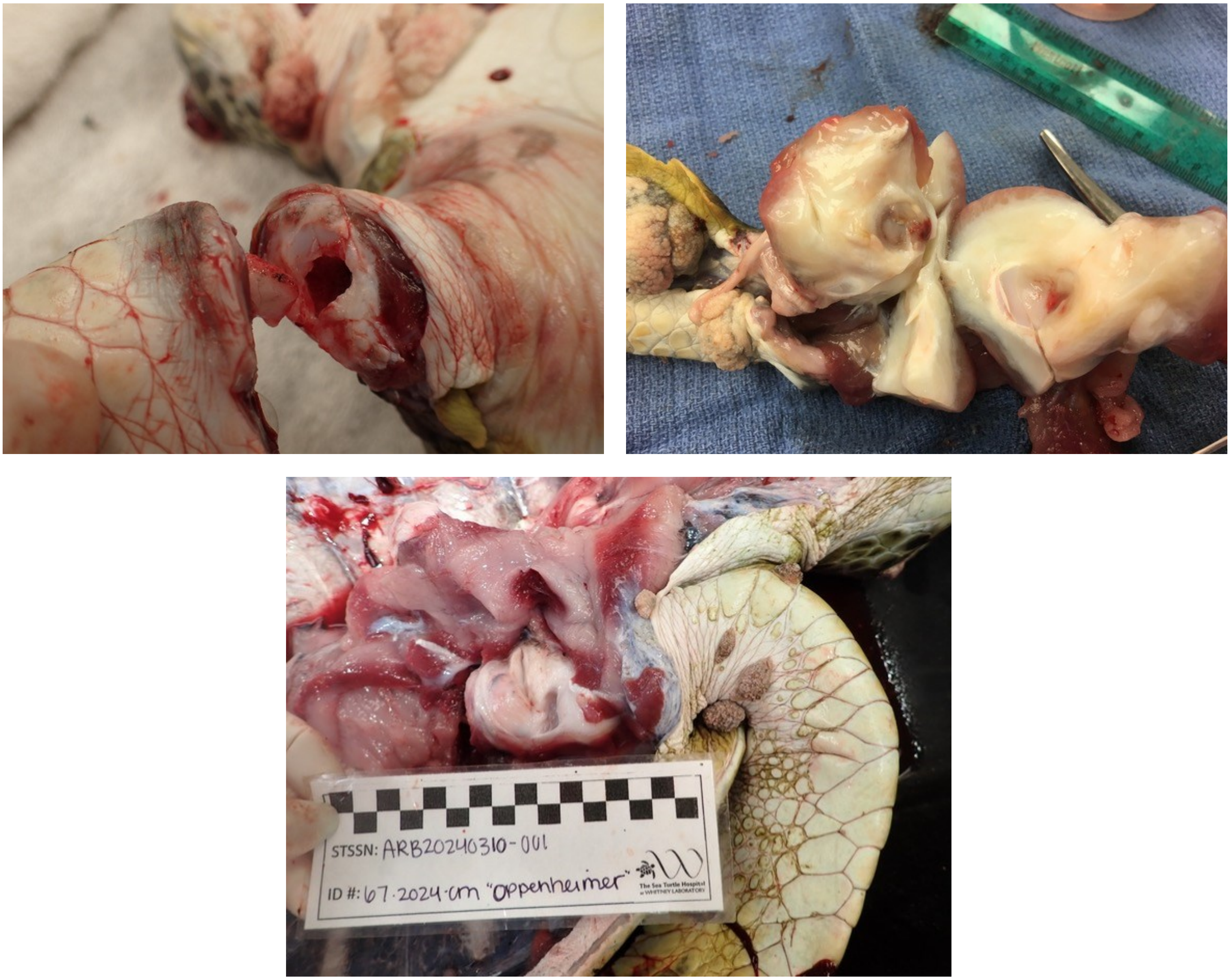

Supplement: Supplementary file 1 [file Presentation1.pdf]
